# Amantadine augmentation in electroconvulsive therapy-resistant catatonia: a case report.

**DOI:** 10.1192/j.eurpsy.2023.2264

**Published:** 2023-07-19

**Authors:** L. Bueno Sanya, H. Andreu Gracia, O. De Juan Viladegut, L. Olivier Mayorga

**Affiliations:** Psychiatry, Hospital Clínic de Barcelona, Barcelona, Spain

## Abstract

**Introduction:**

Catatonia is a syndrome characterized by physical symptoms ranging from immobility to excessive motor activity. Besides being historically associated mainly with schizophrenia, it is widely known that it can be the expression of different psychiatric, neurological or medical conditions. The treatment of choice is benzodiazepines, indicating electroconvulsive therapy in refractory cases. Amantadine is considered a second-line therapy in setting when electroconvulsive therapy is not available.

**Objectives:**

To describe the case of a patient with treatment-resistant catatonic schizophrenia. Not having responded to benzodiazepines or electroconvulsive therapy, potentiation with amantadine was subsequently started.

**Methods:**

Our patient presented at a psychopathological level; psychomotor inhibition, a perplexing attitude, and mute speech. At the motor level; ambitendency, indecision, automatic obedience, motor stereotypes, and facial grimaces. He did not present other alterations at the neurological level. Regarding complementary explorations, we performed neuroimaging and blood tests, which resulted all anodyne. With an alternative diagnosis, we considered that sequelae of a neuroleptic malignant syndrome could have produced the symptoms that he had suffered. Due to the persistence of the symptoms and their typical characteristics, catatonia was our first diagnostical impression. As treatment with benzodiazepines was ineffective, electroconvulsive therapy was started. No clinical improvement was observed. Given the refractoriness of the case, a review of the existing literature was carried out. We found reports of a good response to amantadine in similar cases. Amantadine was introduced up to a dose of 200mg.

**Results:**

After four weeks of treatment with amantadine at a dose of 200mg, the patient showed meager improvement at both psychopathological and motor level.

**Conclusions:**

We find the case of a patient with long-term schizophrenia who is nowadays dependent on all daily living activities and requires sustained care.

**Disclosure of Interest:**

None Declared

